# New *Ent*-Kaurane-Type Diterpene Glycosides and Benzophenone from *Ranunculus muricatus* Linn.

**DOI:** 10.3390/molecules201219801

**Published:** 2015-12-15

**Authors:** Bi-Ling Wu, Hui-Liang Zou, Fang-Min Qin, Hong-Yu Li, Guang-Xiong Zhou

**Affiliations:** Guangdong Province Key Laboratory of Pharmacodynamic Constituents of Traditional Chinese Medicine and New Drugs Research, Institute of Traditional Chinese Medicine and Natural Products, College of Pharmacy Jinan University, Guangzhou 510632, China; Biling5@163.com (B.-L.W.); zouhuiliang@163.com (H.-L.Z.); qinfangmin_77@126.com (F.-M.Q.); hongyu88926@126.com (H.-Y.L.)

**Keywords:** *Ranunculus muricatus*, *ent*-kaurane diterpene, ranunculoside, benzophenone, ranunculone C

## Abstract

Two new *ent*-kaurane diterpene glycosides, ranunculosides A (**1**) and B (**2**), and a new benzophenone, ranunculone C (**3**), were isolated from the aerial part of *Ranunculus muricatus* Linn. The chemical structures of compounds **1**–**3** were established to be (2*S*)-*ent*-kauran-2β-ol-15-en-14-*O*-β-d-glucopyranoside, (2*S,4S*)-*ent*-kauran-2β,18-diol-15-en-14-*O*-β-d-glucopyranoside, and (*R*)-3-[2-(3,4-dihydroxybenzoyl)-4,5-dihydroxy-phenyl]-2-hydroxylpropanoic acid, respectively, by spectroscopic data and chemical methods. The absolute configuration of **1** was determined by the combinational application of RP-HPLC analysis and Mosher’s method.

## 1. Introduction

**Figure 1:**
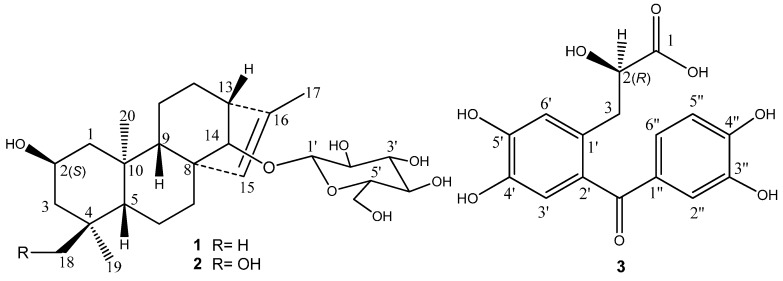
Structures of compounds **1**–**3**.

*Ranunculus muricatus* Linn. (Ranunculaceae) is widely distributed in Asia, South America, Australia, and Europe. This plant mainly grows in the lower Yangtze River regions of China. It is used in folklore medicines in India for the therapy of tonsillitis diseases [1]. Although there is little documentation for folk and clinical use of the titled plant in China, other *Ranunculus* plants such as *R.* *sceleratus* Linn., *R. japonicus* Thunb., and *R.* *termatus* Thunb. have diverse clinical uses with a long history in traditional Chinese medicines and folk medicines, including those used for the treatment of lymphatic tuberculosis, malaria, swollen hemorrhoids, scrofula, and arthritis [2,3,4]. A series of simple lactone derivatives with 5- or 6-member rings, some steroids, and phenols was found in these plants, some of which indicated various biological activities, and they are responsible for their traditional or folk uses and clinical efficacies [5,6]. Our interest is in finding out the chemical characteristics and biological activities of the widely distributed plant *R.* *muricatus*. Wang *et al.*’s research led to the isolation and identification of eight known constituents from this plant, which are stigmasta-4-ene-3,6-dione, stigmasterol, anemonin, sitosterol, protocatechuic aldehyde, protocatechuic acid, and luteoin, and most of which belong to common compounds in plants [7]. Our previous study has revealed the presence of rich flavonoid glycosides in the plant [8]. Now, our further phytochemical investigation on the ethanolic extract from the aerial part of *R.* *muricatus* Linn. led to the isolation and identification of three new compounds (Figure 1), ranunculoside A (**1**), ranunculoside B (**2**), and ranunculone C (**3**). It is notable that ranunculosides A (**1**) and B (**2**) are the first two *ent*-kaurane diterpenoids from the genus plants and are present in glycoside. Herein we describe the isolation and structure elucidation of these compounds through spectroscopic analysis and chemical methods.

## 2. Results and Discussion

The ethanolic extract from the aerial part of *R.* *muricatus* Linn. was successively partitioned in an H_2_O suspension solution with petroleum ether, EtOAc, and *n*-BuOH, then applied to various column chromatography with silica gel, Octadecylsilyl (ODS), and Sephadex LH20, following by preparative reversed phase HPLC purification to yield compounds **1**–**3**.

**Figure 2:**
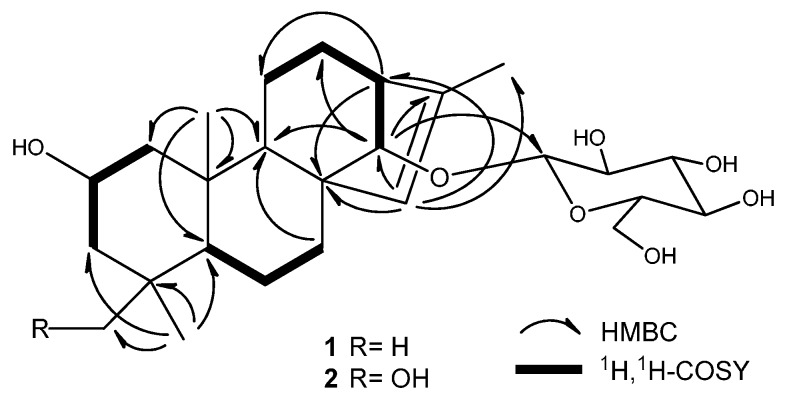
Key HMBC and ^1^H,^1^H-COSY correlations of compounds **1** and **2**.

**Figure 3:**
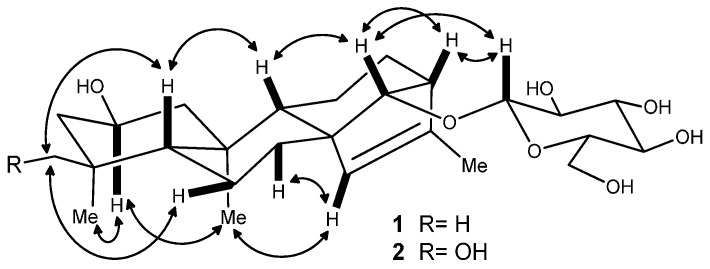
Key NOE correlations of compounds **1** and **2**.

**Figure 4:**
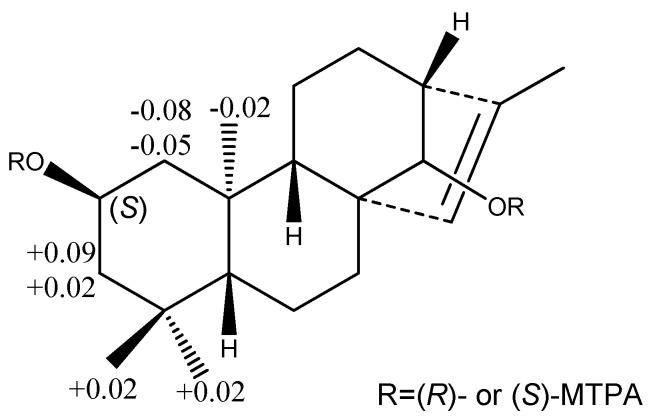
The Δδ_(S-R)_ values of some protons (in C_5_D_5_N, in ppm) obtained from (*S*)- and (*R*)-MTPA esters of compound **1a**.

Compound **1** was obtained as an amorphous solid. Its HR-ESI-MS in positive ion mode showed a pseudomolecular ion peak at *m*/*z* 489.28287 [M + Na]^+^, suggesting its molecular formula as C_26_H_42_O_7_. In the ^1^H-NMR spectrum of **1** (Table 1), there were the signals at δ_H_ 0.83 (s, 3H), 0.86 (s, 3H), 0.87 (s, 3H), and 1.66 (d, *J* = 1.3 Hz, 3H) for four methyl groups. The ^1^H-NMR spectrum displayed signals for the protons of a sugar moiety including a β-oriented anomeric proton signal at δ_H_ 4.88 (d, *J* = 7.8 Hz, 1H), and two other oxygenated methine protons at δ_H_ 4.12 (m, 1H) and 3.78 (br s, 1H) in the range of δ_H_ 3.90–5.00. In addition, there was an olefinic proton signal that appeared at δ_H_ 5.34 (br s, 1H). The ^13^C-NMR spectrum of **1** (Table 2) displayed 26 resonances, including six resonances ascribable to a sugar unit. The distortionless enhancement by polarization transfer (DEPT) experiment revealed that the 26 carbons were comprised of four methyls, seven methylenes, eleven methines, and four quaternary carbons, including a pair of cyclic olefinic carbons at δ_C_ 127.3 and 138.1. The heteronuclear multiple-bond coherence (HMBC) spectrum showed the correlations of protons at δ_H_ 5.34 (br s, H-C(15)) with carbons at δ_C_ 52.8 (s, C(8))/48.8 (d, C(13))/97.7 (d, C(14))/138.1 (s, C(16)), and protons at δ_H_ 3.78 (br s, H-C(14)) with carbons at δ_C_ 32.7 (t, C(7))/53.3 (d, C(9))/127.3 (d, C(15)), suggesting that the olefinic group was located between C(15) and C(16). The OH group at the C(2) position was evident from the correlations between the proton at δ_H_ 4.12 (m, H_α_-C(2)) and protons at δ_H_ (2.20/1.02) (m, CH_2_(1))/(2.03/1.43) (m, CH_2_(3)) in the ^1^H,^1^H-COSY spectrum of **1** (Figure 2). The above data suggested that the aglycone of **1** may be a diterpene (Figure 1). Meanwhile, the correlations of protons at δ_H_ 0.86 (s, Me(18)) with carbons at δ_C_ 52.3 (t, C(3))/35.2 (s, C(4))/55.1 (d, C(5))/23.4 (q, C(19)), and protons at δ(H) 0.83 (s, Me(20)) with carbons at δ_C_ 49.8 (q, C(1))/55.1 (d, C(5))/53.3 (d, C(9))/39.4 (s, C(10)) were observed in the HMBC spectrum, suggesting that the diterpene has *an ent*-kaurane-type skeleton, which was further confirmed to have connections with CH_2_(1)/H-C(2)/CH_2_(3), H-C(5)/CH_2_(6)/CH_2_(7), and H-C(9)/CH_2_(11)/CH_2_(12)/H-C(13)/H-C(14) spin-coupling systems (Figure 2) by the ^1^H, ^1^H-COSY. In the ROESY spectrum of **1**, the explicit nuclear Overhauser effects (NOE) correlations (Figure 3) of protons at δ_H_ 4.12 (m, H_α_-C(2)) with protons at δ_H_ 0.86 (s, Me(19))/0.83 (s, Me(20)) determined the OH group at C(2) to be in β-orientation. Similarly, the NOEs of protons at δ_H_ 3.78 (br s, H-C(14)) with protons at δ_H_ 0.97 (m, H_β_-C(9))/4.88 (d, *J* = 7.8 Hz, H_β_-C(1′))/2.72 (br s, H_β_-C(13)) suggested H-C(14) to be β-orientational. Furthermore, the NOEs described in Figure 3 offered full relative stereochemical information of the diterpene skeleton. The sugar moiety was identified as d-glucopyranose by its ^1^H and ^13^C data and the HPLC analysis of the sugar derivative after the acid hydrolysis of **1** and the successive chemical derivatization of monosaccharide. The connection of the sugar group at C(14) was established by the HMBC correlation between protons at δ_H_ 4.88 (d, *J* = 7.8 Hz, H-C(1′)) and carbons at δ_C_ 97.7 (d, C(14)), and the comparison with the NMR data of pterokaurane M_2_, a very similar molecule with an aglycone in its structure [9]. The absolute configuration of the aglycone (**1a**) in **1** was confirmed by Mosher’s method [10]. Based on the differences of the chemical shifts of diagnosing protons (for Δδ_(S-R)_ values**,** see Figure 4) between the (*R*)-MTPA ester and (*S*)-MTPA ester and the model for determining *R* or *S* configuration, the absolute configuration of acyloxylated carbon (C(2)) in **1a**, then that of **1**, was assigned as *S* by using the diagnostic MTPA ester model. Therefore, the structure of compound **1** was clarified to be (2*S*)-*ent*-kauran-2β-ol-15-en-14-*O*-β-d-glucopyranoside, and named as ranunculoside A.

**Table 1:** ^1^H-NMR Data of **1**, **1a**, and **2** (at 300 MHz, in CD_5_N_5_; δ in ppm, *J* in Hz).

H-Atom	1a (Multiplet)	1 (Multiplet)	2 (Multiplet)
CH_2_(1)	α 2.27 (ddd, *J* = 12.3, 3.8, 2.1)	α 2.20 (brd, *J* = 12.3)	α 2.25 ^o^
	β 1.13 (m)	β 1.02 (m)	β 1.10 (m)
H-C(2)	α 4.16 (m)	α 4.12 (m)	α 4.27 (m)
CH_2_(3)	α 2.05 (ddd, *J* = 12.3, 3.8, 2.1)	α 2.03 (brd, *J* = 12.3)	α 2.16 (m)
	β 1.46 (m)	β 1.43 (m)	β 2.06 (m)
H-C(5)	β 0.88 ^o^	β 0.71 (dd, *J* = 10.6, 1.2)	β 1.52 ^o^
CH_2_(6)	α 1.36 ^o^	α 1.26 ^o^	α 1.50 ^o^
	β 1.61 ^o^	β 1.47 ^o^	β 1.70 ^o^
CH_2_(7)	α 2.33 (dd, *J* = 10.7, 3.2)	α 2.21 (brd, *J* = 10.2)	α 2.29 ^o^
	β 1.62 (m)	β 1.57 (m)	β 1.73 (m)
CH_2_(9)	β 1.09 (m)	β 0.97 (m)	β 1.03 ^o^
CH_2_(11)	α 1.45 ^o^	α 1.38 ^o^	α 1.40 ^o^
	β 1.24 ^o^	β 1.18 ^o^	β 1.23 ^o^
CH_2_(12)	α 1.50 ^o^	α 1.51 ^o^	α 1.52 ^o^
	β 1.32 ^o^	β 1.30 ^o^	β 1.28 ^o^
H-C(13)	β 2.44 (br s)	β 2.72 (br s)	β 2.73 (br s)
H-C(14)	β 3.50 (br s)	β 3.78 (br s)	β 3.75 (br s)
H-C(15)	5.38 (br s)	5.34 (br s)	5.38 (br s)
Me(17)	1.70 (d, *J* = 1.1)	1.66 (d, *J* = 1.3)	1.67 (d, *J* = 1.3)
Me(18)	0.94 (s)	0.87 (s)	a 3.64 (brd, *J* = 10.5)
CH_2_(18)			b 3.43 (brd, *J* = 10.5)
Me(19)	0.92 (s)	0.86 (s)	0.98 (s)
Me(20)	0.90 (s)	0.83 (s)	0.94 (s)
H-C(1′)		β 4.88 (d, *J* = 7.8)	β 4.88 (d, *J* = 7.8)
H-C(2′)		3.95 (m)	3.95 (m)
H-C(3′)		3.98 (m)	3.99 (m)
H-C(4′)		4.28 ^o^	4.26 ^o^
H-C(5′)		4.28 ^o^	4.27 ^o^
CH_2_(6′)		a 4.43 (dd, *J* = 11.7, 5.3)	a 44.4 (dd, *J* = 11.8, 5.3)
		b 4.61 (dd, *J* = 11.7, 2.3)	b 4.59 (dd, *J* = 11.7, 2.4)

Note: (i) ^o^ Overlapped; (ii) Multiplicity: s-singlet, br s-broad singlet; d-doublet, dd-double doublet, ddd-double double doublet, m-multiplet; (iii) α and β mean the orientations of protons; (iv) a and b mean the two protons of a methylene; (v) Assignments were performed by two-dimensional (2D) NMR.

**Table 2:** ^13^C-NMR Data of **1**, **1a**, and **2** (at 75 MHz, in CD_5_N_5_; δ in ppm).

C-Atom	1a	1	2
C(1)	50.1 (t)	49.8 (t)	49.7 (t)
C(2)	64.3 (d)	64.2 (d)	64.5 (d)
C(3)	52.4 (t)	52.3 (t)	46.5 (t)
C(4)	35.3 (s)	35.2 (s)	40.0 (s)
C(5)	55.7 (d)	55.1 (d)	48.7 (d)
C(6)	20.3 (t)	20.0 (t)	20.0 (t)
C(7)	32.8 (t)	32.7 (t)	32.5 (t)
C(8)	54.0 (s)	52.8 (s)	53.0 (s)
C(9)	53.7 (d)	53.3 (d)	53.3 (d)
C(10)	39.5 (s)	39.4 (s)	39.4 (s)
C(11)	19.5 (t)	19.4 (t)	19.5 ^a^ (t)
C(12)	24.0 (t)	23.5 (t)	23.6 (t)
C(13)	52.9 (d)	48.8 (d)	48.6 (d)
C(14)	92.2 (d)	97.7 (d)	97.6 (d)
C(15)	127.4 (d)	127.3 (d)	127.5 (d)
C(16)	138.5 (s)	138.1 (s)	138.0 (s)
C(17)	16.3 (q)	16.2 (q)	16.2 (q)
C(18)	34.3 (q)	34.1 (q)	71.9 (t)
C(19)	23.5 (q)	23.4 (q)	19.5 ^a^ (q)
C(20)	17.0 (q)	16.9 (q)	17.6 (q)
C(1′)		102.9 (d)	102.8 (d)
C(2′)		75.2 (d)	75.2 (d)
C(3′)		78.9 ^a^ (d)	79.0 ^a^ (d)
C(4′)		72.1 (d)	72.2 (d)
C(5′)		78.9 ^a^ (d)	79.0 ^a^ (d)
C(6′)		63.2 (t)	63.3 (t)

Note: ^a^ Overlapped; d indicates CH, q indicates CH_3_, s indicates quaternary carbon, t indicates CH_2_.

Compound **2** was obtained as an amorphous powder, and had a molecular formula of C_26_H_42_O_8_ according to the pseudomolecular ion peak at *m*/*z* 505.27707 [M + Na]^+^ in HR-ESI-MS (positive mode), 16 mass units higher than that of **1**, implying the presence of an additional oxygen atom in **2**. Comparison of the NMR data of **2** with those of **1** indicated that they were a pair of compounds extremely analogous in structure. The mere difference was that the methyl group (Me(18)) in **1** was replaced by a hydroxymethyl group in **2**, because the hydroxymethyl signals at δ_H_ 3.43/3.64 (dd, *J* = 10.5 Hz, 1H each) and δ_C_ 71.9 (t) were observed in the ^1^H- and ^13^C-NMR spectra of **2**. The complete structure of **2** was confirmed by HMBC and NOE correlations, which were similar to those of **1** (Figure 2 and Figure 3). The acid hydrolysis of **2** under the same conditions mentioned in **1** also afforded the same sugar, d-glucose, which was also determined by the comparative HPLC analysis of the sugar derivative with that of standard sugars. Based on the consideration of the same biogeneric pathway, the absolute configurations of all the chiral centers except C-4 in the aglycone part of **2** should be identical to those of **1**. The absolute configuration of the newly present chiral carbon C(4) due to the hydroxyl substitution was determined by careful analysis of its NOESY. Therefore, compound **2** was determined to be (2*S,*4*S*)-*ent*-kauran-2β,18-diol-15-en-14-*O*-β-d-glucopyranoside, which was named ranunculoside B.

**Figure 5:**
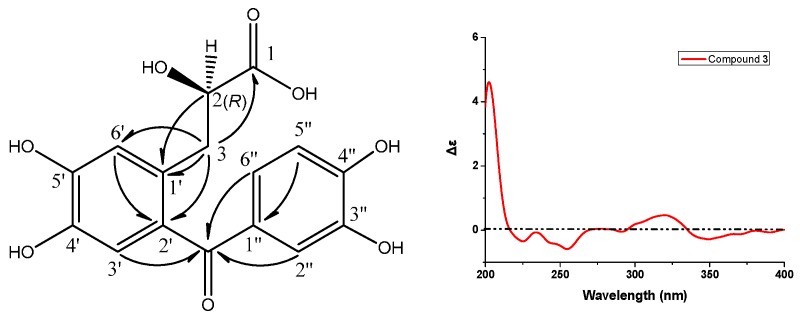
Key HMBC (H → C) correlations and CD spectrum of compound **3**.

Compound **3** showed a sodiated molecular ion peak at *m*/*z* 357.05848 ([M + Na]^+^) in HR-ESI-MS, corresponding to the molecular formula C_16_H_14_O_8_. The ^13^C-NMR spectrum displayed 16 carbon signals, ascribable to two aromatic rings at δ_C_ 115–155 (Table 3), one carbonyl at δ_C_ 199.3, a carboxyl carbon at δ_C_ 177.5, an oxygenated methine at δ_C_ 73.2, and a methylene at δ_C_ 38.0. The ^1^H-NMR spectrum (Table 3) of **3** displayed a AA′X spin system with protons at δ_H_ 2.92 (dd, *J* = 13.8, 8.0 Hz, 1H), 3.12 (dd, *J* = 13.7, 3.8 Hz, 1H), and 4.28 (dd, *J* = 7.8, 3.9 Hz, 1H), which was verified by ^1^H,^1^H-COSY, and five aromatic protons. An *ABX-type* aromatic proton spin system at δ_H_ 7.30 (d, *J* = 1.8 Hz, H–C(2″), 1H), 7.20 (dd, *J* = 8.3, 1.8 Hz, H-C(6″), 1H), and 6.83 (d, *J* = 8.3 Hz, H-C(5″), 1H) was assigned to one tri-substituted benzene ring. Additionally, the other two aromatic protons at δ_H_ 6.81 (br s, H-C(3′), 1H) and 6.88 (br s, H-C(6′), 1H) belonged to the second tetra-substituted benzene ring. The methylene at δ_H_ 2.92/3.12 and C(3) (t, δ_C_ 38.0) was directly connected to oxygenated methine at δ_H_ 4.28/(d, δ_C_ 73.2, C(2)), a chiral center. The HMBC correlations between protons at δ_H_ 2.92/3.12 (CH_2_(3)) and carbon at δ_C_ 177.5 (s, C(1)) suggested that **3** had a 2-hydroxypropanoic acid group. The correlations of protons at δ_H_ 2.92/3.12 (CH_2_(3)) with carbons at δ_C_ 177.5 (C(1))/131.6 (s, C(2′))/119.3 (d, C(6′)) suggested that the 2-hydroxypropanoic acid group connected to the aromatic ring have an *AB-*type spin system. The key correlations of carbon at δ_C_ 199.3 (s, C=O) with protons at δ_H_ 6.81 (s, H-C(3′))/7.30 (s, H-C(2″))/7.20 (s, H-C(6″)) in the HMBC spectrum of **3** (Figure 5) implied its skeleton of diphenylketone. Importantly, other correlations (Figure 5) of H-C(2) with C(1′), H-C(6′) with C(2′), and H-C(5″) with C(1″) also confirmed the above conclusion. Structurally, compound **3** was nearly identical to the reported compound ethyl (*S*)-3-[2-(3,4-dihydroxybenzoyl)-4,5-dihydroxyphenyl]-2-hydroxypropanoate except for the ethanolic esterification at its C(1) in the latter, which had a negative specific rotation and *S* absolute configuration [11]. Noticeably, the specific rotation of **3** is positive, opposite to the reported one above, but consistent with the compound salvianic acid *A* [12] with *R* absolute configuration. Therefore, the only chiral center (d, C(2)) in **3** was discriminated as *R*. According to all the above evidence, compound **3** was identified to be (*R*)-3-[2-(3,4-dihydroxybenzoyl)-4,5-dihydroxyphenyl]-2-hydroxylpropanoic acid, designated as ranunculone C.

**Table 3:** ^1^H- and ^13^C-NMR Data of **3** (at 300/75 MHz for ^1^H/^13^C, in CD_3_OD; δ in ppm, *J* in Hz).

C-Atom	δ(C)	H-Atom	δ(H) Multiplet
C(1)	177.5 (s)		-
C(2)	73.2 (d)	H-C(2)	4.28 (dd, *J* = 7.8, 3.9)
C(3)	38.0 (t)	CH_2_(3)	a 2.92 (dd, *J* = 13.8, 8.0)
			b 3.12 (dd, *J* = 13.7, 3.8)
C=O	199.3 (s)		-
C(1′)	131.0 (s)		-
C(2′)	131.6 (s)		-
C(3′)	118.6 (d)	H-C(3′)	6.81 (br s)
C(4′)	143.9 (s)		-
C(5′)	149.0 (s)		-
C(6′)	119.3 (d)	H-C(6′)	6.88 (br s)
C(1″)	131.4 (s)		-
C(2″)	118.3 (d)	H-C(2″)	7.30 (d, *J* = 1.8)
C(3″)	146.1 (s)		-
C(4″)	152.2 (s)		-
C(5″)	115.6 (d)	H-C(5″)	6.83 (d, *J* = 8.3)
C(6″)	125.9 (d)	H-C(6″)	7.20 (dd, *J* = 8.3, 1.8)

Note: For δ(C), d indicates CH, s indicates quaternary carbon, t indicates CH_2_. - means no correlation to be observed.

All the new compounds were found to be inactive in evaluation for their cytotoxicity against HEp-2 and HeLa cell lines at a test concentration of 50 μg/mL by MTT assay. The procedure was similar to the one in the literature [13], and the data were not presented here.

## 3. Experimental Section

### 3.1. General

TLC: precoated aluminum gel plates (Yantai Chemical Industry Research Institute, Yantai, China). Column Chromatography (CC): silica gel (SiO_2_; 200–300, and 300–400 mesh; Qingdao Marine Chemical Factory, Qingdao, China); D101 macroporous resin (Tianjin Agricultural Pesticide Factory, Tianjin, China); ODS (50 µm, YMC, Tokyo, Japan); Sephadex LH-20 (GE Healthcare Co. Uppsala, Sweden). M.p.: *X-5* micro-melting point apparatus, uncorrected. Optical rotations: Jasco P-1030 automatic digital polarimeter (JASCO Corporation, Tokyo, Japan). UV spectra: Jasco V-550 UV/VIS spectrometer (JASCO Corporation), in anh. MeOH; λ_max_ (log ε) in nm. IR spectra: Jasco FT/IR-480 plus spectrometer (JASCO Corporation), as KBr pellets; in cm^−1^. HR-ESI-MS spectra: Agilent 6210 LC/MSD TOF mass spectrometer (Agilent Technologies, Santa Clara, CA, USA); in *m*/*z* (rel. %). NMR spectra: Bruker AV-300, AV-400 (for data of HMBC), AV-500 (for data of ROESY) instruments (Bruker Corporation, Fallanden, Switzerland), TMS as internal standard, δ in ppm, *J* in Hz. HPLC: Agilent series 1200 (Agilent Technologies) with quaternary pump, multiple wavelength detector and autosampler, and Ultimate™ XB-C18 column (5 µm, 250 × 4.6 mm, or 21.2 × 250 mm, Welch Materials, Inc., Shanghai, China).

### 3.2. Plant Material

The experimental material was gathered in Jiujiang County, Jiangxi Province, China, in July 2010 and authenticated as *Ranunculus muricatus* Linn. by Prof. X.-Z. Zhang, College of Pharmacy, Guangdong Pharmaceutical University. A voucher specimen (No. RM-200927) was deposited in the Department of Pharmacognosy, College of Pharmacy, Jinan University.

### 3.3. Extraction and Isolation

Air-dried and powdered herbal materials (aerial part) of *Ranunculus muricatus* Linn. (10.0 kg) were soaked in 95% alcohol for 24 h and filtered each time, and the process repeated three times. The filtered solution was gathered and evaporated off the alcoholic solvent *in vacuo* to get an extract in about 1.0 kg. The alcoholic extract was suspended in distilled water and then partitioned successively with petroleum ether, ethyl acetate and *n*-BuOH to afford three fractions: petroleum ether fraction (80.0 g), EtOAc fraction (27.1 g), and BuOH fraction (65.2 g), respectively. The EtOAc fraction (27.0 g) was subjected to CC (SiO_2_; PE/EtOAc 30:1 → 0:1), to afford seven pooled fractions (*FrA–G*) according to the TLC pattern. *FrF* (700.0 mg) was applied to CC (SiO_2_; CHCl_3_/MeOH 15:1 → 5:1) to get three subfractions (*SFr 1–3*). *SFr 2* (50.0 mg) was chromatographed on CC (SiO_2_; CHCl_3_/MeOH 8:1) to get *SFr 2-1*, and it was then purified by CC (*sephadex LH-20*; CHCl_3_/MeOH 1:1) to afford **1** (30.3 mg) as an amorphous white powder. The *n*-BuOH fraction (65.0 g) was suspended in water, then applied to *D101 macroporous resin* (EtOH/H_2_O 0:1 → 90:10) to obtain *SFr H-1* (12.2 g), *SFr H-2* (6.2 g), and *SFr H-3* (35.1 g). *SFr H-1* (12.0 g) was applied to CC (*ODS*; MeOH/H_2_O 1:5 → 3:5) to get *SFr H-1-8* (400.0 mg). This fraction was further chromatographed on CC (SiO_2_; CHCl_3_/MeOH 10:1 → 5:1) to get *SFr H-1-8-1*, which was finally purified by CC (*SephadexLH-20*; CHCl_3_/MeOH 1:1) to afford **2** (25.1 mg) as an amorphous white powder. The fraction *SFr H-1-2* (300.0 mg) was applied to CC (*ODS*; MeOH/H_2_O 1:5 → 3:5), and then purified on CC (*Sephadex LH-20*; CHCl_3_/MeOH 1:1) to afford *SFr H-1-2-1* (25.1 mg), which was further purified by preparative HPLC to afford **3** (20.0 mg).

*Acid hydrolysis of **1** and identification of absolute configuration of **1a*** *by Mosher’s method* [9,10]. Compound **1** (9.0 mg) was hydrolyzed with 10 mL of 2 M HCl for 2 h at 90 °C; after cooling down in the air, the mixture was extracted with EtOAc (20 mL) three times. The EtOAc portion was dried *in vacuo*, and applied to CC (SiO_2_; CHCl_3_/acetone 20:1), and finally purified by CC (*Sephadex LH-20*; CHCl_3_/MeOH 1:1) to yield the aglycone of **1** (**1a**, 7 mg) as an amorphous white powder. The aglycone **1a** (1.4 mg) was dissolved in 500 μL of C_5_D_5_N, and then we added (*R*)-(−)-α-methoxy-α-(trifluoromethyl) phenylacetyl chloride (10 μL; Sigma, St. Louis, MO, USA) into it. The Mosher reaction process was protected by nitrogen. The reaction mixture containing the (*R*)-MTPA ester (**1ar**) of **1a** was directly analyzed by NMR. The (*S*)-MTPA ester (**1as**) preparation and analysis was in the same procedure as that of **1ar**. On the basis of the proton chemical shift between **1ar** and **1as**, the differences of the δ values of protons at the same positions were calculated (Figure 4). So the absolute configuration of C(2) was determined.

*Determination of absolute configuration of sugar by RP-HPLC* [14]. The absolute configuration of sugar unit of **1** was discriminated by reversed-phase (RP) HPLC method. Compound **1** (2.0 mg) was hydrolyzed with 2 mL of 1 M HCl for 2 h at 90 °C, and the mixture was dried *in vacuo*. The residue was dissolved in pyridine (1.5 mL) containing l-cysteine methyl ester hydrochloride (1.5 mg; Adamas-beta, Shanghai, China) and heated at 60 °C for 1 h, and then we added *O*-tolylisothiocyanate (5 μL; Sigma) into the mixture and heated it at 60 °C for 1 h. The final reaction mixture was directly analyzed by RP-HPLC. Analytical HPLC was performed on the column at 30 °C with isocratic elution of 25% CH_3_CN (0.8‰ formic acid) for 40 min and subsequently washing the column with 90% CH_3_CN at a flow rate of 0.8 mL/min. The peak signals were detected by a UV detector at 250 nm. The peak of the derivative from **1** was clearly observed at 22.62 min. Standard sugars (Sigma) d-glucose and l-glucose were applied to the same analytical procedure. The peaks of control sugar derivatives were recorded at 22.63 (d-glucose), and 20.75 (l-glucose) min, respectively. The key peak of the derivative from **1** corresponded with the derivative of control d-glucose (t*_R_* 22.63 min), which confirmed the D configuration of the sugar moiety.

The absolute configuration of the sugar moiety of **2** was determined by the same method and analytical procedure as those of **1**. The peak of the derivative from **2** was observed at 21.73 min, which coincided with the derivative of control D-glucose (t*_R_* 21.77 min).

Ranunculoside A (=(*2S*)*-ent-kauran-2*β*-ol-15-en-14-O-*β*-d-glucopyranoside*; **1**): Amorphous white powder; M.p. 230–232 °C; ${\left[\alpha \right]}_{D}^{25}$ = −31.0 (*c* 0.01, MeOH); UV (MeOH) λ_max_ 206 nm; IR (KBr) ν_max_: 3371, 2925, 2852, 1161, 1036 cm^−1^; ^1^H- and ^13^C-NMR data (in C_5_D_5_N), see Table 1 and Table 2; HR-ESI-MS: 489.28287 ([M + Na]^+^, calcd. for C_26_H_42_O_7_Na, 489.28227).

The aglycone (**1a**) of **1**(=*(2S)-ent-2*β*,14*α*-diol-15-en-kauran*; **1a**). Amorphous white powder; M.p. 246–248 °C; ${\left[\alpha \right]}_{D}^{25}$ = −17.2 (*c* 0.05, MeOH); UV (MeOH) λ_max_ 204 nm; IR (KBr) ν_max_: 3367, 2925, 2854, 2360, 1383, 1036 cm^−1^; ^1^H- and ^13^C-NMR data (in C_5_D_5_N), see Table 1 and Table 2; HR-ESI-MS: 327.22969 ([M + Na]^+^, C_20_H_32_NaO_2_^+^; calcd. 327.22945).

Ranunculoside B (=*(2S,4S)-ent-kauran-2*β*,18-diol-15-en-14-O-*β*-d-glucopyranoside*; **2**). Amorphous white powder; M.p. 198–200 °C; ${\left[\alpha \right]}_{D}^{25}$ = −20.4 (*c* 0.05, MeOH); UV (MeOH) λ_max_ 206 nm; IR (KBr) ν_max_: 3371, 2932, 2856, 1169, 1033 cm^−1^; ^1^H- and ^13^C-NMR data (in C_5_D_5_N), see Table 1 and Table 2; HR-ESI-MS: 505.27707 ([M + Na]^+^, C_26_H_42_NaO_8_^+^; calcd. 505.27719).

Ranunculone C (=*(R)-3-[2-(3,4-dihydroxybenzoyl)-4,5-dihydroxyphenyl]-2-hydroxylpropanoic acid*; **3**). Amorphous yellow powder; M.p. 203–205 °C; ${\left[\alpha \right]}_{D}^{25}$ = +7.5 (*c* 0.05, MeOH); UV (MeOH) λ_max_ (log ε): 204 (4.29), 236 (4.00), 287 (3.80), 322 (3.84) nm; CD: 219 nm (Δε-0.17); IR (KBr) ν_max_: 3422 (br s), 1715, 1592, 1521, 1298, 889, 832, 784, 635 cm^−1^; ^1^H- and ^13^C-NMR data (in CD_3_OD), see Table 3; HR-ESI-MS: 357.05848 ([M + Na]^+^, C_16_H_14_NaO_8_^+^; calcd. 357.05809).

## 4. Conclusions

Ranunculosides A (**1**) and B (**2**) are the first two *ent*-kaurane diterpenoids present in glycoside from the genus plants. Up until now, with the exception of *R. ternatus* [11], *R.* *muricatus* is the second *Ranunculus* plant to have benzophenone-type compounds isolated out. All the compounds showed no cytotoxicity against HEp-2 and HeLa cell lines at a test concentration of 50 μg/mL by MTT assay.
